# Role of Telemedicine in Pace of Consultation and Physicians’ Satisfaction in Thoracic Surgery ICU

**Published:** 2018-02

**Authors:** Lida Fadaizadeh, Elham Shajareh, Mohammad Jafar Taheri, Gholamreza Heydari, Behrooz Fazanegan, Marjan Sistani

**Affiliations:** 1 Telemedicine Research Center, National Research Institute of Tuberculosis and Lung Diseases (NRITLD), Shahid Beheshti University of Medical Science, Tehran, Iran; 2 Tobacco Prevention and Control Research Center, NRITLD, Shahid Beheshti University of Medical Sciences, Tehran, Iran; 3 Tracheal Diseases Research Center, NRITLD, Shahid Beheshti University of Medical Sciences, Tehran, Iran

**Keywords:** Tele-consultation, ICU, Physicians’ satisfaction, Delay time, Telemedicine

## Abstract

**Background:**

Despite various applications of tele-ICU, there are still many questions about its costs and advantages in ICU. Some of its advantages are accelerating consultations and bringing physicians’ satisfaction from tele-consultation outcomes. The aim of this study is to discuss these advantages.

**Materials and Methods:**

Initially a telemedicine network was implemented and in the case of having no related specialist, the physicians used telemedicine network to perform specialized tele-consultation to thoracic surgery ICU patients. ICU patient’s documents during a year before tele-consultation were studied and delay time in consultation was recorded and compared between the two phases. Finally, the physicians’ satisfaction with tele-consultation was evaluated.

**Results:**

Fifty-eight tele-consultations in various medical fields were carried out, of which 27 were neurology cases. From the time of receiving a consultation request to its performance, the mean time was 1.3 days in tele-consultation. Tele-consultations were given 2.5 times faster than face to face method. In evaluation of physicians’ satisfaction, 82.75% of them were fully satisfied from tele-consultation, 12.06% were partly satisfied and 5.17% were not satisfied.

**Conclusion:**

Since the length of hospitalization in ICU is crucial due to heavy costs of treatment, high risk of contamination and limited beds, performing timely consultation is a key factor in reducing hospitalization period. Tele-consultation in thoracic surgery ICU not only accelerates patient care, but also results in higher physician satisfaction.

## INTRODUCTION

Tele-ICU is the use of advanced communication technologies by implementing a network in which a team of off-site physicians is connected with distant ICU patients to make clinical decisions.

Since the ICU patients are mostly complicated and cannot be mobilized, these patients usually suffer multiple organ disorders and it is essential to have the consultation of various specialists, usually off-site, to make proper decisions. Therefore, telemedicine can be considered an appropriate solution.

Some advantages of using telemedicine in consultation are decrease in the rate of transferring patients to other centers, accelerating patient management, decreasing side effects, and thus shortening the length of hospitalization (1,2). Moreover, using tele-ICU for the sickest patients is not only cost-effective (3); it can also promote the quality of services in ICU.

Telemedicine in ICU has been studied in recent decades, but there are still many unanswered questions regarding the benefits of its application (4). Some advantages of telemedicine are accelerating consultations and bringing physicians’ satisfaction from outcomes, especially in high risk thoracic surgery patients, which will be discussed in this study.

## MATERIALS AND METHODS

This study was performed in a tertiary pulmonology/thoracic surgery hospital as a cross sectional study. Initially, a communication network for specialized consultations was established among seven specialized hospitals affiliated to Shahid Beheshti University of Medical Sciences. Fiber optic communication; and web conference software (Microsoft Link) was used for simultaneous audio-visual connection (webcam (Microsoft lifecam HD-3000**)**. Tele-examination devices and equipment were provided including camera for examining ear, eye and skin (Dino-Lite (AM4113/AD4113)) and also digital stethoscope (JABES analyzer) for heart and lung examination.

Patients selected for tele-consultation were mostly complicated thoracic surgery cases with multiple organ failure for whom transportation was not only infeasible, but also contraindicated. Also due to critical situation of patients, timely performance of consultation and reaching a conclusion was a necessity. The consulting physician was located in a remote hospital and long distance transportation was required to perform the visit. For tele-consultation, initially the necessary instructions were given to the project team, including physicians, nurses and IT personnel, on how to operate the system. The physician would provide all necessary documents (the patient’s history, tests, ECG, radiology documents) and transfer to consultant physician via store and forward, and then discuss the case online via videoconference. It is noteworthy that only patients who had signed the consent letter for tele-consultation participated in the study.

To compare the pace of tele-consultation and regular (bedside) consultation the documents of ICU patients admitted during the year before starting tele-ICU were studied. In these cases the mean time between requesting consultation and visit by the off-site physicians was evaluated. Then this interval (mean time between requesting and answering the consultation) was compared between the two groups using Mann- Whitney non-parametric test.

Finally, the physicians’ satisfaction from tele-consultation was assessed using a questionnaire comprising of three choices: fully satisfied, partly satisfied, and not satisfied. Full satisfaction was achieved when the consultations were given on-time, with desired results and effective in improving treatment. However, when there was a problem in consultation procedure but finally the requesting physician could get the desired result, he/she was partly satisfied. No satisfaction was declares when the requesting physician did not get any answer to the consultation.

## RESULTS

Fifty-eight consultations were performed in various specialized fields. All patients had undergone thoracic surgery procedures, were critically ill with multiple organ failure and impossible to transfer and timely consultation was required for prompt patient management. The highest rate of consultation was in neurology (27 cases) and thereafter, in neurosurgery (11 cases). Other consultations were in endocrinology (3 cases), gastroenterology (2 cases), thoracic surgery (2 cases), vascular surgery (2 cases), ophthalmology (1 case), hematology (1 case) and dermatology (1 case).

Among the 58 participants there were 23 female patients and 35 male patients. The mean age of patients was 61.32 years. Consultations were given from October 2013 to December 2015 (26 months) and the mean number of monthly consultations was 2.23 cases.

Mean time between requesting and performing a tele-consultation was 1.3 days while document review of ICU patients during a year before starting tele-ICU showed that this period was 3.3 days (Table 1). Therefore; tele-consultations were answered 2.5 times faster. Mann-Whitney nonparametric test showed a statistically significant difference between the two values (p-value< 0.05).

**Table 1.** Evaluation of rapidity in giving tele-consultation

**Consultation time**	**The same day in**	**The day after**	**2 days later**	**3 days later**
Neurology	6	21	-	-
Neurosurgery	5	4	-	2
Endocrinology	1	2	-	-
Gastroenterology	-	2	-	-
Orthopedics	-	1	1	-
Urology	1	3	-	-
Gynecology	2	-	-	-
Thoracic surgery	-	2	-	-
Vascular surgery	3	-	-	-
Ophthalmology	1	-	-	-
Hematology	-	-	-	1
Dermatology	-	1	-	-
Total consultations	18(%)	36(%)	1(%)	3(%)

Satisfaction survey results showed that the physicians were fully satisfied with tele-consultations in 82.75% of cases. They were partly satisfied in 12.06% and not satisfied in 5.17% of consultations (Table 2).

**Table 2.** Evaluation of physicians’ satisfaction from tele-consultation

**Consultation field**	**Fully Satisfied**	**Partly satisfied**	**Not satisfied**
Neurology	26	1	-
Neurosurgery	9	2	-
Endocrinology	3	-	-
Gastroenterology	2	-	-
orthopedics	-	1	1
Urology	4	-	-
Gynecology	-	1	1
Thoracic surgery	-	2	-
Vascular surgery	2	-	-
Ophthalmology	1	-	-
Hematology	-	-	1
Dermatology	1	-	-
Total consultations	48(82.75%)	7(12.06%)	3(5.17%)

No major problem related to connection, network or devices occurred during the consultations.

## DISCUSSION

Results of the present study indicated the effectiveness of telemedicine in accelerating off-site consultations and therefore decision making in thoracic surgery ICU patients, with high physician satisfaction. Also the efficacy of Shahid Beheshti University of Medical Sciences tele-medicine network in performing ICU tele-consultations was demonstrated.

Shortening the length of hospitalization in ICU is substantial due to heavy costs of treatment, high risk of contamination and limited beds. This length depends on different factors one of which is providing quick consultation and therefore prompting decision making for the patients. Especially when the consultant physician is off-site and the patient cannot be transferred due to his/her unstable conditions, much time will be wasted for answering the consultation due to the physician’s work load and therefore delay in visiting the patient. Considering this importance, this study evaluated the quickness of answering to tele-consultation. It showed the fact that tele-consultations were given 2.5 times faster than face to face consultations which result in quicker decision making and eventually shortening the length of stay. This is mostly important in patients with special situations such as those who have undergone major thoracic surgeries, are prone to various complications and therefore require specialized management.

Several other studies have considered the use of telemedicine time saving, for instance a study was done in 2013 that compared the use of telemedicine method and regular method for ICU patients aged younger than six weeks. The study revealed that telemedicine shortened the time to diagnosis and it significantly decreased the transport of infants between hospitals. Besides, the length of stay and intensive care stay were shortened in telemedicine program (5). Other studies showed that the rate of mortality was significantly shortened in a telemedicine program compared to the control subjects (2,6, 7).

Despite all advantages of using tele-ICU, if the technology is not accepted and used by ICU staff, especially the physician, it will not affect the patient care procedure (8). Considering the fact that the first step in effectiveness of telemedicine is achieved by collaboration of health care staff, satisfaction from tele-consultation can encourage them for further collaboration (9). For example a study which was done in 2012 suggested that tele-ICU improved satisfaction of critical care nursing team, especially during night time hours and enhanced communication among components of ICU team (10).

Another descriptive study reported the implementation of telemedicine in a Dutch intensive care unit and its outcomes including patients’ and their families’ satisfaction. The study suggested that implementation of tele-ICU was successful and desirable results regarding patients’ outcomes and satisfaction of them and their families were seen (11).

Many studies have shown that even consultations between ICU nurses were helpful. For instance in 2012 a study showed that tele-consultation between nurses of different hospitals promoted the quality of support and that inter-hospital collaboration helped improve patient care. Therefore, uses of tele-ICU have no limitations and a wide range of ICU workers benefit from this new method of patient management. (12).

All these studies revealed various benefits of tele-consultation without considering physician satisfaction as an effective factor in its utilization. Studies already conducted deal with nurses’, patients’ and their families’ level of satisfaction but our study deals with physician’s level of satisfaction from tele-consultation in ICU, which we found very high (82.75%). Considering the fact that main decision makers regarding patient management are the physicians, who are actually the end users of this system, makes their opinion very much important for implementing it. On the other hand there is still a global deficit in the number of physicians in charge of ICU patients, and since educating them for the purpose is both time consuming and expensive, it seems more practical for them to take the responsibility of more than one ICU at the same time. For this to happen, the American Telemedicine Association (ATA) has published a guideline clarifying different aspects of this system. In this guideline it has been mentioned that each licensed ICU physician can be in charge of 100–250 patients, and all this can happen by using tele-ICU systems (13). This emphasizes that the more these physicians become familiar with tele-ICU systems and the more they have satisfaction in using it, the wider the range of system utilization and eventually the more the ICU patients receive specialized management.

There are still many unanswered questions about the best way of implementing telemedicine in health care system of our country and therefore carrying out comprehensive studies is essential, but there is no doubt that if this happens, higher quality of patient care and satisfaction will result.

## CONCLUSION

Physician’s level of satisfaction and also the speed of responding to tele-consultations are the two important factors in rendering tele-consultations in ICU and it is anticipated that tele-consultation can be successfully implemented in our ICU’s in the near future. It seems that implementing tele-ICU in hospitals might provide higher quality health services with proper pace and therefore progress of patient management in ICU is also anticipated.

## References

[1] Turk E Karagulle E Aydogan C Oguz H Tarim A Karakayali H Use of telemedicine and telephone consultation in decision-making and follow-up of burn patients: Initial experience from two burn units. Burns 2011;37(3):415–9. 21146313 10.1016/j.burns.2010.10.004

[2] Rosenfeld BA Dorman T Breslow MJ Pronovost P Jenckes M Zhang N Intensive care unit telemedicine: alternate paradigm for providing continuous intensivist care. Crit Care Med 2000;28(12):3925–31. 11153637 10.1097/00003246-200012000-00034

[3] Franzini L Sail KR Thomas EJ Wueste L. Costs and cost-effectiveness of a telemedicine intensive care unit program in 6 intensive care units in a large health care system. J Crit Care 2011;26(3):329.e1–6. 10.1016/j.jcrc.2010.12.004 PMC3103644 21376515

[4] Sapirstein A Lone N Latif A Fackler J Pronovost PJ. Tele ICU: paradox or panacea? Best Pract Res Clin Anaesthesiol 2009;23(1):115–26. 19449620 10.1016/j.bpa.2009.02.001

[5] Webb CL Waugh CL Grigsby J Busenbark D Berdusis K Sahn DJ Impact of telemedicine on hospital transport, length of stay, and medical outcomes in infants with suspected heart disease: a multicenter study. J Am Soc Echocardiogr 2013;26(9):1090–8. 23860093 10.1016/j.echo.2013.05.018

[6] Hawkins HA Lilly CM Kaster DA Groves RH Jr Khurana H. ICU Telemedicine Comanagement Methods and Length of Stay. Chest 2016;150(2):314–9. 27048869 10.1016/j.chest.2016.03.030

[7] Lilly CM McLaughlin JM Zhao H Baker SP Cody S Irwin RS A multicenter study of ICU telemedicine reengineering of adult critical care. Chest 2014;145(3):500–507. 24306581 10.1378/chest.13-1973

[8] Khunlertkit A Carayon P. Contributions of tele-intensive care unit (Tele-ICU) technology to quality of care and patient safety. J Crit Care 2013;28(3):315.e1–12. 10.1016/j.jcrc.2012.10.005 23159139

[9] Ekeland AG Bowes A Flottorp S. Effectiveness of telemedicine: a systematic review of reviews. Int J Med Inform 2010;79(11):736–71. 20884286 10.1016/j.ijmedinf.2010.08.006

[10] Rincon F Vibbert M Childs V Fry R Caliguri D Urtecho J Implementation of a model of robotic tele-presence (RTP) in the neuro-ICU: effect on critical care nursing team satisfaction. Neurocrit Care 2012;17(1):97–101. 22547040 10.1007/s12028-012-9712-2

[11] van der Voort PH de Metz J Wester JP van Stijn I Feijen HM Balzereit A Telemedicine in a Dutch intensive care unit: A descriptive study of the first results. J Telemed Telecare 2016;22(3):141–7. 26141722 10.1177/1357633X15590751

[12] Anders SH Woods DD Schweikhart S Ebright P Patterson E. The effects of health information technology change over time: a study of Tele-ICU functions. Appl Clin Inform 2012; 3(2): 239–47. 23646073 10.4338/ACI-2011-12-RA-0073 PMC3613018

[13] Davis TM. Guidelines for Tele-ICU Operations. www.americantelemed.org. 5 2014

